# A Comprehensive Strategy for Laser Corneal Refractive Surgery during the COVID-19 Epidemic in a Tertiary Teaching Hospital in Wenzhou, China

**DOI:** 10.1155/2020/4835630

**Published:** 2020-07-15

**Authors:** Jia Zhang, Ioannis M. Aslanides, Vasileios Selimis, Nan-Ji Lu, Wei-Jie Liu, Hong-Xiao Jiang, Chao Zhang, Chen-Chen Xu, Qin-Mei Wang, Jia Qu, Shi-Hao Chen

**Affiliations:** ^1^School of Ophthalmology and Optometry and Eye Hospital, Wenzhou Medical University, Wenzhou, Zhejiang, China; ^2^State Key Laboratory of Optometry, Ophthalmology and Vision Science, Wenzhou, Zhejiang, China; ^3^Emmetropia Mediterranean Eye Institute, Heraklion, Greece; ^4^School of Medicine and Health Sciences, University of Antwerp, Wilrijk, Belgium

## Abstract

The novel coronavirus pneumonia COVID-19 is caused by the novel coronavirus SARS-CoV-2, which is highly contagious, has a long incubation period, and can be detected in patients' tears and conjunctival secretions. In this study, we describe our experience regarding the necessary protective measures that need to be taken during ophthalmic examination and treatment. The authors reviewed the clinical work arrangements during the epidemic situation at the Eye Hospital of Wenzhou Medical University in China and analyzed the prevention and control measures that were applied during the laser corneal refractive surgery process. The comprehensive protection protocol, which was established throughout the entire process, included both horizontal (medical staff-patient, medical staff-medical staff, and patient-patient) and vertical (preoperative, intraoperative, and postoperative transmission assessment) approach and was mainly focused on strengthening the protection against potential aerosol transmission that may occur during intraocular pressure measurements and laser ablation. The described and proposed protocol, along with the further guidelines followed by the medical personnel, proved to be efficacious and contributed significantly to the control of the COVID-19 outbreak and the protection of both the patients and the medical staff.

## 1. Introduction

In December 2019, a new type of coronavirus pneumonia (novel coronavirus pneumonia, NCP) epidemic occurred in Wuhan, China. With the large number of people moving during the Spring Festival, the epidemic spread quickly to all parts of the country. About 180,000 people in Wenzhou, Zhejiang Province (China), were doing business and studying in Wuhan. Therefore, at the end of January, the number of cumulative confirmed cases in Wenzhou ranked in the top five nationwide. Since the first week of March, the epidemic situation in China has been effectively controlled, but the number of cases worldwide is increasing (Italy ranks in the top three). The first imported case in Zhejiang Province occurred on 1st March. As of 24 : 00 on March 12, a total of 10 cases have been imported (data source: official website of the Health Commission of Zhejiang Province http://www.zjwjw.gov.cn/art/2020/3/12/art_1202194_42230730.html), all of which had returned from Italy. As Wenzhou is a famous hometown of overseas Chinese, there are about 100,000 Chinese living in Italy. Therefore, we still cannot slacken to prevent and control the spread of the novel coronavirus in imported cases.

The novel coronavirus (also known as severe acute respiratory syndrome-related coronavirus 2, SARS-CoV-2), previously known by the provisional name 2019-nCoV, is highly contagious, has a long incubation period, and can cause a severe respiratory disease known as coronavirus disease 2019 (COVID-19). Conjunctivitis has also been diagnosed in patients positive to SARS-CoV-2 [1–3], and SARS-CoV-2 can be detected in tears and conjunctival secretions in patients with COVID-19 [4], although there is no evidence that the virus can cause disease directly through the conjunctiva on humans. Still, the virus may pass through the nasolacrimal duct system and perhaps cause conjunctivitis while it is possible to be transmitted by aerosol contact with the conjunctiva; however, a study showed that rhesus macaques can be effectively infected with the novel virus via conjunctival route [5]. Since ophthalmologists have a high probability of contacting patients with tears and conjunctival secretions, protection is necessary.

## 2. Review of Outpatient and Surgery during the Epidemic

Every year before the Spring Festival, the laser corneal refractive surgery is at its peak. Our center carried out surgery until January 23, 2020 (the previous day of Spring Festival holiday). On the same day, Zhejiang Province launched the first-level response to the major public health emergency. In accordance with the epidemic situation and the government's prevention and control instructions, combined with a series of hospital new regulations, the outpatient and surgical work was adjusted accordingly.

**Figure 1:**
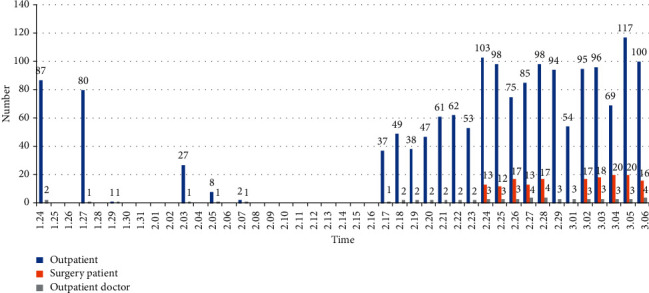
Data of outpatient doctors, patients, and surgical patients in the center (from January 24 to March 6, 2020). Note: the hospital officially resumed work on 17th February.

It can be seen from Figure 1 that before the hospital officially resumed work (February 17), the center significantly reduced the number of outpatients and doctors and canceled surgeries with a relatively high risk of infection. The postoperative follow-up schedule was changed several times after the holidays, and every patient who had already made an appointment was contacted by phone calls or text messages. As laser corneal refractive surgery requires the routine use of steroid eye drops postoperatively, containing dexamethasone, for their anti-inflammatory effects, especially after transepithelial photorefractive keratectomy (TransPRK, TPRK), it is necessary to measure the intraocular pressure to determine if it is within normal limits. Therefore, under conditions of adequate protection, the center has conducted limited follow-up examinations (visual acuity, intraocular pressure, and slit lamp evaluation).

After resuming work, the number of outpatient doctors and scheduled appointments was increased accordingly, but the number of patients remained limited (up to 4 doctors per day, and each doctor has a maximum of 20 appointment numbers for half a day). At the same time, the noon (12 : 00–13 : 30 pm) clinic was canceled, as ultraviolet disinfection of all examination rooms and consultation rooms was performed at that time. Regarding surgical arrangements, priority was given to patients requiring visual examinations, such as candidates of police and military academies, and civil servants while surgeries for other patients were postponed (no more than 10 to 15 in half a day). In order to diversify the number of surgical patients, our center plans to carry out surgeries on Saturday and Sunday to reduce the average daily operation volume and the staff turnover in the operating room.

During the epidemic, the center responded to the hospital's suggestion that all doctors open remote outpatient services to facilitate consultation for patients with moving/traveling problems and issued online prescriptions, which were delivered to the patients' homes after being approved by our hospital pharmacists. After resuming work, the safety education and online counseling services for the surgical patients are specific, and it is recommended that the patients should be checked at the nearest hospital branches to reduce the risk of cross-infection during travel.

In order to avoid clustering, training sessions related to SARS-CoV-2 demonstrated protective measures and multiple ophthalmic academic meetings were held online during the epidemic.

## 3. Prevention and Control Measures

Although the domestic epidemic situation has been basically controlled, in order to prevent the transmission of SARS-CoV-2 in imported cases and asymptomatic infections, preventive and control measures should continue to be enforced until further notice. A number of medical institutions and committees have put forward recommendations for protective measures for ophthalmologists, disinfection methods of equipment, devices, and the environment, and patient management [6–10]. Based on the standard protection of ophthalmology, our center has combined the characteristics of laser corneal refractive surgery and its examination to establish a horizontal and vertical protection protocol. Horizontal protection refers to the protection between medical staff and patients, medical staff and medical staff, and patients and patients, while vertical protection refers to the protection during the three stages of surgery, i.e., the pre-, intra-, and postoperative stages. Horizontal protection was carried out in every period of longitudinal protection.

COVID-19 is mainly caused by the invasion of SARS-CoV-2 in the respiratory tract mucosa. Spreading through respiratory droplets and close contact is the main route of transmission. SARS-CoV-2 can remain viable and infectious in aerosol form for at least 3 hours [11], and there might be an aerosol transmission in long-term exposure to high concentrations of aerosol in a relatively closed environment [12]. During noncontact IOP measurement, aerosols will gather around the air outlet and will have a cumulative effect with the increase of the number of jets [13, 14], and the density of aerosols in the environment with insufficient air circulation will be higher [14]. During excimer laser ablation of corneal tissue (including part of the tear fluid), a large number of tissue particles are formed containing respirable particles in the plume [15], especially during the TPRK procedure and the surface ablation of the cornea. Those particles can enter the respiratory tract and surface on the conjunctiva. The risk of infection may be further increased in a closed operating room space with no laminar flow. Therefore, in the prevention and control measures, the aerosol propagation that may occur during intraocular pressure measurement and laser ablation cannot be ignored, and it is necessary to strengthen the protection and strive to cut off all possible transmission routes of SARS-CoV-2.

### 3.1. Outpatient Procedure and Prevention/Control Measures

Corneal thickness is one of the indispensable examinations before corneal refractive surgery. In the A-ultrasound method, the probe needs to contact the corneal surface. In order to avoid cross-infection, it is recommended to use noncontact optical measurement methods, including anterior segment optical coherence tomographic scanner (anterior segment optical coherence tomography (AS-OCT)), corneal topography based on the Scheimpflug principle, optical biometer based on the principle of optical low coherence reflectometry (OLCR), etc.

The direct ophthalmoscope examination is closer to the patient, and there is no isolation device between the doctor and the patient during the binocular indirect ophthalmoscope examination. The center prefers to use a slit lamp indirect ophthalmoscopy lens instead. Fundus photography, such as ultra-widefield ophthalmoscope, can also be used to help to diagnose myopic retinopathy such as peripheral retinal degeneration and holes if it is difficult to observe clearly with goggles.

### 3.2. Surgical Procedures and Prevention and Control Measures

Surgery (especially major surgery) may accelerate and exacerbate disease progression of COVID-19 in asymptomatic patients, and the risk factors for poor prognosis may be old age, comorbidities, surgical time, and difficulty of operation, so the possibility of the new coronavirus infection being excluded before elective surgery is very important [16].

The combined test of serum amyloid A (SAA), C reactive protein (CRP), and blood routine can improve the effectiveness of diagnosis and differential diagnosis of bacterial and viral infections [17]. Eosinopenia combined with elevated CRP could also be used to facilitate the rapid identification of highly suspected COVID-19 patients [18]. In order to avoid the transmission of recessive asymptomatic infections and to reduce the number of nosocomial infections risk, two more tests (SAA and CRP) have been conducted to patients planned for surgery in addition to the routine preoperative blood examinations [19]. Any abnormality of the blood lymphocyte, the leukocyte, and eosinophil is recommended to be further evaluated. The test results could change quickly because of the progression of COVID-19, and as a consequence, we concerned the CRP, SAA, and blood routine as valid only for two days.

After arriving at the center on the surgery day, the patient needed to fill out and sign the epidemiological questionnaire (Annex S1). The importance of truthfully filling out the questionnaire was emphasized to the patients and their families. In the meantime, they were all informed that, according to the Criminal Law and the COVID-19-related law, concealing any relative report can be investigated for legal liability related to the crime of obstructing the prevention and control of infectious diseases. Refer to Annex S2 for the procedure of isolation and observation of patients with fever.

Preoperative examinations of refractive error and conversations were conducted in the outpatient examination room.

Table 1 provides outpatient prevention and control measures.

Table 2 shows surgical procedures and control measures.

## 4. Environmental Disinfection

Stop using central air conditioning. Ultraviolet (1.5 W/m^3^) disinfection should be performed in the examination room and the consultation room twice a day (morning and noon) for 60 minutes each time [21]; 500–1000 mg/L chlorine-containing disinfectant (excluding chlorhexidine) should be used for surface and floor disinfection for 30 minutes, twice a day [22, 23], once before and once after the clinic. Ultraviolet disinfection of the operating room 3 times a day (morning, noon, and night) should be performed. Postoperatively, the walls and floor of the operation room should be sprayed with chlorine disinfectant before wiping. The equipment should be wiped with chlorine disinfectant. Since SARS-CoV-2 can be isolated in feces and urine, attention should be paid to aerosols or contact transmission caused by fecal and urine pollution to the environment [12, 24], and the environment should be disinfected meticulously.

With comprehensive prevention and control measures throughout our hospital, we took the lead in restoring the normal order of diagnosis and treatment in the country. In future times, epidemic prevention and control must be always done with due care, and we need to do our best in protecting patients and doctors in each possible way and carry out the clinical work of laser refractive surgery safely until the epidemic is over.

## Figures and Tables

**Table 1:** Main outpatient procedures and prevention/control measures.

Main process	The basic principle	Precautions	Medical staff	Patients and family members
Triage	① The entrance criteria of the hospital are strictly controlled: admission on the day of the appointment, health code (Annex 3) green, breathing mask compliance, body temperature <37.3°C, up to one companion, and conduct the epidemiological investigation. To those with no issues, colored signs are put on the clothes. (The colors can be different every day, and the effective time is marked.) The patient firstly goes through the epidemiological investigation and the details are entered in the electronic system of the outpatient clinic when he/she comes to the clinic. ② Refer to Annex 2 for the procedure of isolation and observation of patients with fever. ③ Open windows for ventilation during examination and consultation. ④ One patient, one doctor in one room. A family member can accompany if the patient is under the age of 18. ⑤ Everyone and everything gets sterilized: 75% alcohol cotton ball for the disinfection of instruments and appliances (such as test frames, slit lamp indirect ophthalmoscopy lens) where the patient touched (forehead bar, chin rest, etc.), hands-free disinfection solution to disinfect hands, follow “seven-step handwashing” after contact with tears or conjunctival secretions. ⑥ Guide the patients to remain scattered (keep distances with each other) and the family members to wait in a ventilated place, and set up conspicuous signs on the ground, isolation strips, seats, etc. to remind them to be at least 1.5 meters apart. ⑦ Medical staff scattered to have meals at different times and measure their temperature twice a day to report.	① In the epidemiological survey, a question was added asking “whether the patient or the people close to the patient is overseas Chinese or just returned from abroad.” ② Cotton gauze and respirators with noncompliant valves should be replaced in time.	① Wear a five-piece suit: surgical masks, hats, goggles, gloves, and work clothes. ② Slit lamp microscope shield installed to isolate droplets. ③ Wear a face shield when measuring intraocular pressure. ④ Disinfect the face shield and goggles before and after use. Antifog methods for eye masks include antibacterial hand sanitizer, soap-based detergent, iodophor, and antifogging agent [20].	① Wear a breathing mask (other protective equipment can be provided). ② The waiting area is equipped with disposable hand sanitizer.
Outpatient examination and consultation		① All inspections are noncontact (referring to noncontact eye surface). Use a slit lamp indirect ophthalmoscopy lens to exam the fundus instead of a direct ophthalmoscope. ② Strengthened protective measures for noncontact tonometry include installing air barriers with isolation baffles; immediately after the measurement, use 75% alcohol spray to disinfect the air near the tonometer's air outlet and wipe the air outlet; and extend the measurement interval between patients.		
Consulting room schedule an operation		—		

**Table 2:** Main surgical procedures and prevention and control measures.

Main process	Basic principles	Precautions	Medical staff	Patients
Lacrimal tract irrigation, conjunctival sac irrigation, palpebral margin/eye skin/facial skin disinfection	① Take the body temperature, replace the breathing mask by a new one provided by the hospital at the entrance of the operating room, take off the shoes, wear shoe covers, and give a surgical gown to the patient. ② Ventilation during operation. ③ The patients should be at least 1.5 meters apart when waiting. ④ Lacrimal tract flushes one person, one needle, and one tube; the conjunctival sac flushes the eyewash pot, and the treatment towel is changed one by one.	① Immediately after flushing the lacrimal duct and conjunctival sac, use 75% alcohol spray to disinfect the air in the operating space. ② A laminar flow operating room is preferred. If there is no laminar flow system, the air sterilizer can be used to set the cycle work and combined with mechanical ventilation (such as electric fans, medical staff is on the upper air outlet). ③ Shorten the use time of the filter nozzle of the particle absorption system of the laser equipment and disinfect the laser exit with ultraviolet rays after the operation.	① Medical N95 mask, hat, goggles, gloves, disposable surgical gown. Fully closed goggles should be worn in the operating room. ② Wear a face shield during lacrimal tract irrigation and conjunctival sac irrigation. ③ Face shield and goggles should be disinfected before and after use.	Wear a hat before disinfection. Disinfect palpebral margin first, then eye skin and facial skin. (When disinfecting the facial skin, the breathing mask is pulled down between the mouth and nose.)
During surgery	① Change sterilization gloves, a set of instruments, a tablecloth, and a handle cover for each person. ② Aseptic operation. ③ One in and one out. After one surgical patient goes out, the next patient enters again. ④ Strictly control the total number of operating rooms. ⑤ After the slit lamp microscopic examination, the forehead and mandibular support were eliminated by one person.			① Remove the breathing mask and put it in a special bag. After disinfecting the face for the second time, immediately lay on the operating table and put on a sterile hole towel. ② After the operation, wear the breathing mask immediately after getting up from the operating table.
After surgery	① Terminal disinfection. ② Strictly sort and place and manage medical waste.			① Wear postoperative goggles. ② Pay attention to eye hygiene and avoid rubbing eyes and injuries.

## Data Availability

The data used to support the findings of this study are available from the corresponding author upon request.

## Supplementary Materials

Supplementary Materials S1 Annex1. Refractive Surgery Center, Eye Hospital, Wenzhou Medical University Patients' Epidemiological Survey Signing Form, S2 Annex2. IsSolation and observation process for fever patients in the outpatient department of Eye Hospital of Wenzhou Medical University, S3 Annex3. A bBrief introduction to health QR-codes.
