# Supplementary material for: A Comprehensive Strategy for Laser Corneal Refractive Surgery during the COVID-19 Epidemic in a Tertiary Teaching Hospital in Wenzhou, China
Source: J Ophthalmol. 2020 Jul 15;2020:4835630. doi: 10.1155/2020/4835630 (PMC7366208; doi:10.1155/2020/4835630)
Supplement: Supplementary Materials — S1 Annex1. Refractive Surgery Center, Eye Hospital, Wenzhou Medical University Patients' Epidemiological Survey Signing Form, S2 Annex2. IsSolation and observation process for fever patients in the outpatient department of Eye Hospital of Wenzhou Medical University, S3 Annex3. A bBrief introduction to health QR-codes. [file 4835630.f1.docx]

**Supporting information captions**

**S1 Annex1. Refractive Surgery Center, Eye Hospital, Wenzhou Medical University Patients’ Epidemiological Survey Signing Form**

**S2 Annex2. Isolation and observation process for fever patients in the outpatient department of Eye Hospital of Wenzhou Medical University**

**S3 Annex3. A brief introduction to health QR-codes**

**Annex1：**

Refractive Surgery Center, Eye Hospital, Wenzhou Medical University

Patients’ Epidemiological Survey Signing Form

Name ____________________Number of accompanying persons

ID number ______________________ Body temperature ___________________

Blood Regular ______________________ CRP + SAA _____________________

According to the "Notice on Further Improving the Prevention and Control of Pneumonia of New Coronavirus Infection" in Wenzhou, in order to ensure the safety of you and of the personnel, please carefully cooperate with the following:

1. In the past 14 days, are there any people in Hubei Province, Wuhan and surrounding areas, or other communities with case reports?

Travel history: □ Yes □ No

Residence history: □ Yes □ No

2. In the past 14 days, if you have any contact history with new coronavirus infection (positive nucleic acid test) or suspected patient:

□ Yes □ No

3. In the past 14 days, whether you have contact history with patients with fever or respiratory symptoms from Hubei Province, Wuhan City and surrounding areas, or from communities with case reports:

□ Yes □ No

4. Does the contacted family, relatives and friends have the above situation:

□ Yes □ No

5. Are there any other suspicious symptoms such as fatigue, cough, stuffy nose, runny nose, abdominal pain, diarrhea, conjunctivitis, etc.:

□ Yes □ No

6. Whether I and my companion are overseas Chinese or have just returned from abroad:

□ Yes Country □ No

7. Color of my health code: □ green □ yellow □ red

Family health code color: □ green □ yellow □ red

**Tips: If the body temperature is higher than 37.3 ℃, it is recommended that the operation be postponed.**

**Please fill in the above information truthfully, conceal and provide false information, you will bear legal responsibility, thank you for your cooperation.**

**Patient / Guardian (Signature): _________**

**Family temperature: __________ Family (signed): _________**

**Survey period: _________ month _________ day in 2020**

**Annex 2：**

Isolation and observation process for fever patients in the outpatient department of Eye Hospital of Wenzhou Medical University

1. The outpatient doctor or nurse found that the patient had fever (body temperature > 37.3 ° C), chest tightness, or asthma.

2. Inform the security guard to send them to the temporary observation room (set a fixed room).

3. The security guard is at the door.

4. After half an hour, the nurses at the temperature monitoring points (from the temperature monitoring points in the respective buildings) will take the patient's temperature (ear temperature or mouth temperature) again.

5. If the body temperature is normal for two consecutive measurements, chest tightness and asthma improve, continue to return to the original consultation room; if the body temperature is still higher than normal, the nurse fills out a fever patient information form, and the patient wears a surgical mask, hat, disposable isolation gown, sent by the security guard to the fever clinic of a general hospital.

**Annex 3：**

A brief introduction to health QR-codes

Health QR-codes play a digitized public management tool in work resumption and economy recovery. It creates conducive conditions for the Chinese people in epidemic control and resumption of work, which are both crucial to win the battle against the virus. Based on pilot practice in Zhejiang since Feb 11, this health code system has been an effective method in national unified epidemic prevention and control. “Health QR-codes” are based on real data including individual and big data. Every citizen can ask for the code through their own online application such as Alipay or WeChat. After reviewing their background based on their shopping, travelling and other online information, they can get their own individual dynamic QR-code. The QR-codes are divided in three types with a corresponding color for each one: green, yellow and red. The green code means people have little chance of having been infected and are allowed to travel freely, while those with yellow code must be held under quarantine for 7 days and with a red one for 14 days, and should report their health information every day. Therefore, the health QR-code serves as an electronic voucher for individuals. It not only provides a rapid, convenient and contactless channel for personal data checkout, which is crucial to preventing the spread of the novel coronavirus, but also helps authorities monitor the epidemic situation and effectively prevent and control its spread. In hospitals, especially in the outpatient department, scanning or checking health QR-codes can improve the efficiency and avoid contact and gathering of people.
